# Fascial Closure and the Occurrence of Port-Site Hernia After Laparoscopic Surgery: A Systematic Review and Meta-Analysis

**DOI:** 10.3390/jcm15218280

**Published:** 2026-10-24

**Authors:** Dimitrios Balafoutas, Konstantinos Stavridis, Panagiotis Christopoulos, Panagiotis Vakas, Nikos Vlahos

**Affiliations:** 1 2nd Department of Obstetrics and Gynecology, “Aretaieion” University Hospital, 115 28 Athens, Greece; stavridis.kost@gmail.com (K.S.); dr_christopoulos@yahoo.gr (P.C.); panagiotisvakas124@yahoo.gr (P.V.); nikosvlahos@med.uoa.gr (N.V.); 2 Department of Obstetrics and Gynecology, Spital Männedorf, 8708 Männedorf, Switzerland

**Keywords:** minimally invasive surgery, port, incisional hernia, laparoscopic surgery

## Abstract

**Background/Objectives**: Port-site hernia (PSH) is a known complication of laparoscopic and robotic surgery, but whether routine fascial closure reduces this risk remains unclear. We examined the association between fascial closure and PSH compared with non-closure. **Methods**: This systematic review and meta-analysis followed PRISMA and was registered in PROSPERO (CRD420251274471). Embase, MEDLINE, APA PsycInfo, Global Health, and HMIC were searched for English-language studies up to 7 March 2026. Randomized trials and comparative observational studies reporting PSH after fascial closure versus non-closure were included. Non-comparative studies and studies without extractable PSH data were excluded. We used random-effects meta-analysis, RoB 2 and ROBINS-I for risk of bias, and GRADE to assess certainty of evidence. **Results**: Ten studies including 2183 patients (3 randomized trials and 7 observational studies) met the inclusion criteria. Overall, fascial closure was not associated with a statistically significant reduction in PSH (RR 0.51, 95% CI 0.25–1.07; I^2^ = 72%). The findings remained non-significant when randomized trials (RR 0.81, 95% CI 0.15–4.38) and observational studies (RR 0.45, 95% CI 0.19–1.06) were considered separately. In a post hoc analysis limited to studies with balanced follow-up, PSH was less frequent after closure (RR 0.38, 95% CI 0.20–0.73). Four observational studies were at serious risk of bias, and certainty of evidence ranged from very low to low. **Conclusions**: The available evidence does not provide a clear answer on whether routine fascial closure reduces PSH. No included study evaluated robotic procedures. More randomized studies with consistent closure techniques and adequate follow-up are needed.

## 1. Introduction

Laparoscopic and robotic surgery are now routine across gynecology, urology and general surgery, giving patients less postoperative pain, quicker recovery, and shorter hospital stays [1,2]. As these platforms have spread—robotic systems typically working through 8-mm ports—the range of what can be done has widened, but access-related complications have not gone away. Port-site hernia (PSH) is among the most debated of them [3,4]. Reported PSH incidence varies considerably across surgical specialties, trocar sizes and types, and detection methods. Clinically detected rates have historically ranged from 0.6 to 5.2% [5], whereas studies using systematic imaging have reported incidences of 8.3% after gynecological laparoscopy [5] and up to 34% after bariatric surgery [3]. These differences highlight the importance of surgical context and surveillance methods when interpreting PSH incidence. Several factors drive the problem. Most hernias form at defects of 10 mm or more, a relationship Kadar described decades ago in gynecologic laparoscopy, where the rate climbed from 0.2% overall to 3.1% once 12-mm trocars were used [6]. Most also cluster at the umbilicus and midline, where the linea alba is weakest [5,7]. Widening the fascial incision to retrieve a specimen, a bulky specimen, long operating times, wound infection, and obesity each add further risk [5,8,9]. The earlier literature on trocar-site hernias highlighted the importance of trocar diameter and fascial closure [10], while the European Hernia Society guidelines recommended closure of trocar sites ≥ 10 mm [11]. The updated joint guidelines of the European Hernia Society and American Hernia Society similarly suggest fascial closure of trocar sites ≥ 10 mm, particularly at the umbilicus and following single-incision laparoscopic surgery. However, this is a weak recommendation based on very-low-certainty evidence [12].

That advice is not universally followed. Surgeons who prefer non-bladed and radially expanding trocars argue that a muscle-splitting entry leaves a defect small enough to leave unrepaired [13,14,15]. The comparative data, however, are mixed: some series find no benefit from closure, and a few even report more hernias in the closed arm [2,3]. Whether routine closure is necessary, particularly for 8-mm robotic and 12-mm bladeless ports, remains uncertain.

We therefore undertook this systematic review and meta-analysis to ask whether fascial closure influences the incidence of PSH in adults undergoing laparoscopic or robotic surgery.

## 2. Materials and Methods

### 2.1. Protocol

The study protocol was registered with the International Prospective Register of Systematic Reviews (PROSPERO; registration number CRD420251274471) before data extraction commenced. The literature search had been completed before registration, and no changes were made to the primary outcome following registration. A minor amendment to the eligibility criteria made after registration is described below.

### 2.2. Information Sources and Search Strategy

The systematic review was conducted according to the guidelines of the Preferred Reporting Items of Systematic Reviews and Meta-analyses (PRISMA) [16]. We performed a literature search for relevant studies across five databases: Embase (OVID), MEDLINE (OVID), APA PsycInfo (OVID), Global Health (OVID), and Health Management Information Consortium (HMIC) (OVID). The database search was conducted using English-language search terms and covered publications up to 7 March 2026. No language or human-study filters were applied during the database search. The following search strategy was used in OVID: (port site hernia or trocar site hernia or incision site hernia or fascial defect or Richter hernia or fascial closure).mp. AND (Laparoscopy or Robotic or da Vinci or Surgical Instruments or Bladeless or 8 mm or radially expanding).mp. The complete electronic search strategy, including field codes and the number of records retrieved at each step, is provided in Appendix A Table A1. To maximize sensitivity, the reference lists of all included studies and pertinent reviews were hand-searched and forward-citation tracking (“snowballing”) was undertaken. Only published studies were sought; investigators were not contacted for unpublished data. The PRISMA 2020 checklist is provided in the Supplementary Materials (Table S1).

### 2.3. Eligibility Criteria

Eligibility was defined a priori using a PICOS framework. The population comprised adults (≥18 years) undergoing laparoscopic or robotic abdominal surgery of any specialty, including general, gynecologic, urologic, and bariatric procedures. The intervention of interest was fascial closure of the trocar-site defect, achieved by suture or by a dedicated fascial closure device; the comparator was non-closure, in which only the skin was approximated. The primary outcome was the incidence of port-site hernia (PSH), diagnosed clinically and/or by radiological imaging and reported separately for each study arm. Pre-specified secondary outcomes were wound infection, fascial dehiscence, bowel obstruction, postoperative pain, and operative time. Both randomized controlled trials (RCTs) and comparative observational studies were eligible.

Studies were excluded if they enrolled pediatric patients (<18 years); involved open surgery, natural-orifice transluminal endoscopic surgery, or single-incision laparoscopic surgery without an active multiport comparator; addressed pre-existing hernias or hernia repair at the trocar site; or did not report PSH data extractable per arm. Narrative reviews, meta-analyses, and case reports were also excluded. Screening was conducted using English-language titles and abstracts, and only human studies were eligible for inclusion. No language restrictions were applied at the database-search stage.

### 2.4. Study Selection

The identification, screening, and selection of studies were conducted and reported in strict adherence to the PRISMA 2020 statement [16], and the review protocol was prospectively registered with PROSPERO (CRD420251274471). Records retrieved from the electronic databases were collated and de-duplicated prior to screening. After removal of duplicate citations, 1060 unique records were screened. To maximize methodological rigor and minimize the premature exclusion of potentially eligible comparative studies, title and abstract screening was performed independently by the two reviewers (D.B and K.S) across two consecutive rounds, with any disagreement resolved by discussion until consensus was reached. On the basis of this two-stage screening, 985 records were excluded as clearly irrelevant to the research question, leaving 75 reports that were sought for retrieval and assessed in full text against the pre-specified eligibility criteria.

Of the 75 full-text articles assessed for eligibility, 66 were excluded, with the reason for exclusion documented for every report. The reasons were as follows: no direct comparison between fascial closure and non-closure (n = 36); a different intervention, most commonly prophylactic mesh placement (n = 12); no extractable data on port-site hernia incidence per study arm (n = 8); ineligible publication type, namely narrative reviews, meta-analyses, or case reports (n = 7); and incomplete or unreadable text precluding reliable data extraction (n = 3). Nine studies identified through database searching fulfilled all eligibility criteria. One additional eligible study was identified through citation searching, resulting in a total of 10 studies included in the systematic review, of which eight contributed to the primary meta-analysis. The full selection pathway, with the corresponding counts at each stage, is presented in the PRISMA 2020 flow diagram (Figure 1). Following full-text assessment, a minor amendment to the eligibility criteria was made to include the randomized trial by Yacoub et al., which enrolled participants aged ≥ 14 years. This decision was made before data extraction because adult-only data were not available, and the randomized design was considered to provide important comparative evidence. The amendment was documented after PROSPERO registration and is reported transparently.

### 2.5. Data Extraction and Data Items

Data were extracted by one reviewer using a standardized, piloted data-collection form and were independently verified by the second reviewer, with disagreements resolved by consensus. Corresponding authors were not contacted, and only published data were used. For each study, the following were recorded: first author, year, country, and study design; surgical specialty and procedure; surgical approach (conventional laparoscopy vs. robotic); the number of patients in the closure and non-closure arms; baseline age and BMI, where reported; trocar size and type (bladed, non-bladed/dilating, or radially expanding); anatomical port and specimen-extraction location (umbilical/midline vs. lateral/off-midline); the method and duration of PSH ascertainment (clinical examination, ultrasonography, or computed tomography); the number of PSH events per arm; and the pre-specified secondary outcomes, where reported. Where quantitative pooling was not feasible, the corresponding data were synthesized narratively.

### 2.6. Risk-of-Bias Assessment

Two reviewers (D.B and K.S) independently appraised the methodological quality of each included study, with disagreements resolved by consensus. Randomized trials were assessed with the Cochrane Risk of Bias 2 (RoB-2) tool across its five domains—yielding an overall judgment of low risk, some concerns, or high risk [17]. Observational studies were assessed with the Risk Of Bias In Non-randomized Studies of Interventions (ROBINS-I) tool across its seven domains, yielding an overall judgment of low, moderate, serious, or critical risk [18]. Risk-of-bias assessment figures were created via the web-app robvis (Risk-Of-Bias VISualization) [19]. Overall, grading the quality of evidence was assessed by the Grading of Recommendations, Assessment, Development, and Evaluation (GRADE) framework [20].

### 2.7. Statistical Analysis

Baseline age and BMI were summarized descriptively using aggregate study-level data. For studies reporting group-specific means, standard deviations, and sample sizes, aggregate means were calculated using sample-size weighting. Aggregate standard deviations were reconstructed by combining within-study variance and between-study differences in group means. These estimates were used exclusively for descriptive purposes, without inferential comparisons, because individual participant data were unavailable and observations were clustered within studies.

Quantitative synthesis was performed in Review Manager Web (RevMan Web version 11.9.0). The primary outcome was pooled as a risk ratio (RR) with corresponding 95% confidence intervals (CIs) using an inverse-variance random-effects model. A random-effects approach was selected because clinical and methodological heterogeneity between studies was anticipated. Between-study variance (τ^2^) was estimated using the DerSimonian–Laird method. To assess the robustness of the findings to the choice of variance estimator, an additional sensitivity analysis was performed using restricted maximum likelihood (REML) estimation. A further post hoc sensitivity analysis excluded Yacoub et al. [4] to assess the influence of the amendment to the originally registered age eligibility criterion (≥18 years). Statistical heterogeneity was assessed using Cochran’s Q test and quantified with the I^2^ statistic. An additional post hoc sensitivity analysis including double-zero-event studies was performed using a continuity correction of 0.5 and a DerSimonian–Laird random-effects model. Analyses were conducted using the R package meta through Posit Cloud (RStudio).

To explore heterogeneity and assess the robustness of the pooled estimate, we performed a subgroup analysis stratified by study design (RCTs versus observational studies) and a post hoc sensitivity analysis excluding studies with a documented marked between-group difference in follow-up duration. Such differences may introduce differential outcome ascertainment for the time-dependent outcome of port-site hernia. Studies reporting a common follow-up period for both groups, or without evidence of differential follow-up, were retained. In response to the substantial heterogeneity observed in the primary analysis, an additional exploratory post hoc subgroup analysis was conducted according to surgical setting (bariatric versus non-bariatric surgery), including both randomized and observational studies. Subgroup differences were assessed using a formal test for interaction. All statistical tests were two-sided, with *p* < 0.05 considered statistically significant, including tests for subgroup differences. Subgroup analyses were considered exploratory and interpreted cautiously, taking into account the limited number of studies and the potential for spurious findings. *p* values are reported to a maximum of three decimal places in accordance with journal style.

## 3. Results

### 3.1. Study Selection

Ten studies involving 2183 patients (933 in the fascial closure group and 1250 in the non-closure group) were included in the systematic review. Eight studies contributed to the primary meta-analysis, while two were non-estimable because no port-site hernias occurred in either treatment group. The study selection process is presented in Figure 1.

### 3.2. Study Characteristics

The 10 studies were published between 2002 and 2025 and came from eight countries across Europe, Asia, North America, and Africa. Three were randomized controlled trials [1,2,4]; the remaining seven were comparative observational studies [3,5,8,9,15,21,22]. Sample sizes ran from 40 to 359 patients. The surgical settings were mixed: four studies were bariatric [3,4,8,9], one benign gynecologic laparoscopy [5], one colorectal resection [21], one laparoscopic renal surgery [15], and three general-laparoscopic caseloads [1,2,22]. The methods used to detect port-site hernias varied considerably across studies: some studies relied on clinical examination alone [15,22], others on routine ultrasound [3,5,8] or computed tomography [9,21], and follow-up ranged from roughly 5 months to more than 4 years. Age and BMI were summarized descriptively from studies reporting sufficient group-level data. The reconstructed aggregate mean age was 42.8 ± 16.1 years in the closure group and 45.5 ± 16.2 years in the non-closure group, while the corresponding BMI values were 41.9 ± 11.8 and 41.3 ± 11.8 kg/m^2^ (Figure A1). These estimates were derived from study-level summaries rather than individual patient data and should not be interpreted as formal between-group comparisons. All ten studies used conventional laparoscopy; although robotic procedures were eligible, no comparative robotic study met the inclusion criteria. Table 1A,B summarize these characteristics.

### 3.3. Risk of Bias

Among the three randomized trials appraised with RoB-2, two were judged to be at overall low risk of bias [1,4], while one raised some concerns [2], arising principally from the randomization process, missing outcome data, and selection of the reported result. Across the seven observational studies appraised with ROBINS-I, the overall risk of bias was serious in four [8,9,15,21] and moderate in three [3,5,22]. The serious judgments were driven predominantly by the confounding domain, as none of the observational studies adjusted adequately for the differential indications that determined whether a fascia was closed—with the selection-of-participants domain contributing additional concern. The domain-level and overall risk-of-bias assessments are presented in the traffic-light plots (Figure 2 and Figure 3), while the corresponding summary plots are provided in Appendix A (Figure A4 and Figure A5). Detailed domain-level ROBINS-I judgments and study-specific justifications are provided in Table A2 (Appendix A).

Overall, the certainty of the evidence according to the GRADE approach ranged from very low to low. The certainty of evidence from randomized controlled trials was rated as low because of very serious imprecision: only 13 events occurred among 332 participants, and the confidence interval (RR 0.81, 95% CI 0.15–4.38) encompassed substantial benefit and potential harm. The certainty of evidence from observational studies was rated as very low because of serious risk of bias and serious inconsistency across the included studies (Table 2).

### 3.4. Meta-Analysis

Of the 10 included studies, eight provided estimable relative risks for the primary meta-analysis. The remaining two studies, by Shalhav et al. [15] and Singal et al. [22], reported no port-site hernias in either group. Although these studies were retained in the systematic review, they did not contribute to the pooled relative-risk estimate because a relative risk cannot be calculated directly when both groups have zero events. This was a limitation of the statistical approach rather than a reason to exclude these studies from the review. The primary analysis was inconclusive. Although the pooled point estimate favoured fascial closure, the confidence interval included no effect (RR 0.51, 95% CI 0.25–1.07; *p* = 0.07), and substantial between-study heterogeneity was observed (I^2^ = 72%) (Figure 4). Sensitivity analysis using REML estimation yielded a comparable pooled effect (RR 0.51, 95% CI 0.26–1.01; *p* = 0.055; I^2^ = 67%), without changing the statistical interpretation of the primary analysis. Including all 10 studies with a continuity correction of 0.5 yielded results comparable to the primary analysis (RR 0.53, 95% CI 0.27–1.05; I^2^ = 63.7%), without changing the overall interpretation (Figure A2).

In accordance with Cochrane guidance [23], studies were stratified by design to evaluate the pooled effect within each group (Figure 5). Among the randomized controlled trials, fascial closure was not associated with a significant reduction in PSH (RR 0.81, 95% CI 0.15–4.38; *p* = 0.80; I^2^ = 42%). Among the observational studies, fascial closure showed a trend towards a reduced risk of PSH; however, the pooled effect did not reach statistical significance (RR 0.45, 95% CI 0.19–1.06; *p* = 0.07), with considerable residual heterogeneity (I^2^ = 81%). Although the magnitude of the effect appeared greater in the observational studies than in the randomized trials, the test for subgroup differences was not statistically significant (*p* = 0.54), suggesting that study design did not explain the observed between-study heterogeneity. An additional exploratory post hoc subgroup analysis was conducted according to surgical setting (bariatric versus non-bariatric surgery), including both randomized and observational studies (Figure A3). The pooled RR was 0.62 (95% CI 0.31–1.23; I^2^ = 60%) among bariatric studies and 0.52 (95% CI 0.11–2.33; I^2^ = 60%) among non-bariatric studies. No statistically significant subgroup difference was observed (*p* = 0.83), and substantial heterogeneity persisted within both subgroups, suggesting that surgical setting did not adequately explain the heterogeneity observed in the primary analysis.

### 3.5. Sensitivity Analysis

We performed a post hoc sensitivity analysis to explore whether differences in follow-up duration between treatment groups influenced the pooled findings. As in the primary analysis, the two studies reporting no hernia events in either group were retained in the review but did not contribute to the relative-risk estimate. This analysis was undertaken because substantial differences in follow-up duration may introduce differential outcome ascertainment for the time-dependent outcome of port-site hernia. Karampinis et al. was excluded because follow-up was markedly longer in the fascial closure group than in the non-closure group (81 ± 20 vs. 27 ± 18 months), potentially biasing hernia detection. Among the seven studies contributing estimable risk ratios after exclusion of Karampinis et al., between-study heterogeneity was reduced (I^2^ = 33%; χ^2^ = 8.94, df = 6, *p* = 0.18), and fascial closure was associated with a statistically significant reduction in the risk of port-site hernia (RR 0.38, 95% CI 0.20–0.73; *p* = 0.003) (Figure 6). As this was a post hoc exploratory analysis, these findings should be interpreted cautiously. The observed reduction in heterogeneity does not establish follow-up imbalance as its cause. Future updates of this review should prespecify criteria for assessing and handling differences in follow-up duration between treatment groups. An additional post hoc sensitivity analysis excluding Yacoub et al. yielded a pooled RR of 0.55 (95% CI 0.25–1.18; *p* = 0.13; I^2^ = 75%) (Figure A7). This was similar to the primary analysis (RR 0.51, 95% CI 0.25–1.07; I^2^ = 72%), indicating that inclusion of this trial did not materially affect the pooled findings or their inconclusive interpretation.

### 3.6. Publication Bias

Given that fewer than 10 studies contributed to the meta-analysis, funnel-plot assessment and formal tests for funnel-plot asymmetry were not performed because of their limited reliability in meta-analyses with few studies, in accordance with Cochrane guidance.

### 3.7. Secondary Outcomes

Secondary outcomes were reported inconsistently and were therefore synthesized narratively. Surgical site infection was reported comparatively by Gupta et al. [1], Lago et al. [2], Yacoub et al. [4], Ece et al. [8], and Singal et al. [22], with no statistically significant differences between fascial closure and non-closure. More specifically, Yacoub et al. reported infection in 5/35 versus 4/35 patients (*p* = 0.721) [4], while Ece et al. reported 8/146 versus 11/206 cases [8]. Operative time was reported by Krittiyanitsakun et al. [21], Karampinis et al. [3], Yacoub et al. [4], Ece et al. [8], Shalhav et al. [15], and Rebibo et al. [9]; most studies found longer operative times with fascial closure, including 170 versus 123 min in Krittiyanitsakun et al. (*p* = 0.004) [21] and 166 versus 110 min in Karampinis et al. (*p* < 0.001) [3], whereas Rebibo et al. reported shorter operative time in the closure cohort (58 versus 90.2 min; *p* < 0.001) [9]. Postoperative pain was assessed by Yacoub et al. [4], Krittiyanitsakun et al. [21], and Shalhav et al. [15]; Yacoub et al. reported markedly more moderate or severe pain following closure (*p* < 0.001) [4], and Krittiyanitsakun et al. likewise found lower postoperative pain with non-closure (*p* = 0.018) [21], whereas Shalhav et al. found no significant difference in analgesic requirements (*p* = 0.52) [15]. Bleeding was uncommon and showed no significant differences where reported. Overall, the available secondary-outcome data showed no consistent difference in perioperative morbidity. Although some studies reported longer operative times with fascial closure and two studies reported greater early postoperative pain, differences in surgical procedures, study designs, and outcome reporting limit the interpretation of these findings. Acute bowel incarceration was rarely reported; Ece et al. described one incarcerated bowel loop, while no comparative pattern could be established because of the scarcity of events [8] (Table 3).

## 4. Discussion

This systematic review and meta-analysis evaluates the association between fascial closure and port-site hernia, incorporating formal assessments of risk of bias and certainty of evidence.

The primary analysis was inconclusive. While the pooled relative risk favoured fascial closure (RR 0.51), the confidence interval included no effect and between-study heterogeneity was substantial. Greater interpretative weight should be given to the randomized evidence because of its lower susceptibility to confounding. However, the three randomized trials provided highly imprecise results (RR 0.81, 95% CI 0.15–4.38), with few hernia events and a confidence interval encompassing both substantial benefit and potential harm. The observational studies also favoured fascial closure (RR 0.45, 95% CI 0.19–1.06), but their findings were limited by potential confounding and risk of bias. Therefore, the overall pooled estimate should not be interpreted as stronger evidence of a treatment effect than the randomized trials alone. An exploratory post hoc sensitivity analysis excluding the study with markedly imbalanced follow-up between groups yielded a lower pooled risk estimate (RR 0.38, 95% CI 0.20–0.73; I^2^ = 33%). However, because this analysis was not prespecified, its findings should be considered hypothesis-generating and do not alter the inconclusive result of the primary analysis. It suggests that differences in follow-up may have contributed to the observed heterogeneity, but further studies are needed to confirm this. Overall, these findings suggest that methodological differences between studies may explain much of the observed heterogeneity and are broadly consistent with current European and American Hernia Society recommendations supporting closure of trocar defects ≥ 10 mm, while acknowledging that the certainty of the supporting evidence remains low [12].

Although fascial closure was not associated with consistent differences in surgical site infection or bleeding, five studies reported longer operative times, with between-group differences ranging from 6 to 56 min, while one study reported a 32.2-min shorter duration [3,4,8,9,15,21]. These differences may affect operating-room efficiency but cannot be attributed solely to fascial closure, as total operative time is influenced by procedural complexity and patient characteristics. Furthermore, two studies reported greater early postoperative pain following closure [4,21], although its clinical magnitude and duration remain uncertain because of inconsistent reporting.

Three recent meta-analyses have examined fascial closure and port-site hernia, with differing conclusions. Al-Mufargi et al. included six studies involving 1066 patients and reported a significant reduction in hernia risk following closure (RR 0.38, 95% CI 0.23–0.63), although the absolute risk difference was small and not statistically significant [24]. In contrast, Abou Hussein et al. and Zhu and Liu focused on bariatric surgery and found insufficient evidence to support routine closure [25,26]. These differences may partly reflect variations in surgical populations, study selection, and analytical methods. Our review included several surgical specialties, assessed randomized and observational studies separately, and considered risk of bias, certainty of evidence, and differences in follow-up. These methodological factors may help explain why the available reviews have reached different conclusions.

Two studies appeared to account for most of the observed heterogeneity. Among the included studies, Karampinis et al. warrants particular methodological consideration because the fascial closure group underwent substantially longer follow-up than the non-closure group (81 ± 20 vs. 27 ± 18 months) [3]. Given that port-site hernia is a time-dependent outcome, this imbalance may have increased hernia detection in the fascial closure group independently of the true effect of the intervention, thereby introducing differential outcome ascertainment. Although this methodological limitation does not invalidate the study, it provides a plausible explanation for part of the observed heterogeneity and highlights the importance of standardized follow-up and outcome assessment in future trials. Lago et al. was the only randomized trial to report more hernias following fascial closure; however, this finding was based on very few events and should be interpreted cautiously [2]. An additional exploratory subgroup analysis investigated whether surgical setting contributed to the observed heterogeneity. Stratification into bariatric and non-bariatric studies revealed no significant subgroup difference (*p* = 0.83), while substantial heterogeneity persisted within both subgroups (I^2^ = 60%). These findings suggest that surgical setting alone did not adequately account for the variability in treatment effects.

Evidence regarding perioperative outcomes remains limited, as most studies primarily assessed port-site hernia. Although no consistent differences in infection or bleeding were observed [4,15,22], postoperative pain and operative time may vary according to the closure technique and device used [4,8,9,15,27,28]. Given the inconsistent reporting of these outcomes, firm conclusions cannot be drawn.

Although a clinically relevant benefit of fascial closure remains possible, the current evidence is insufficient to establish its magnitude with confidence. A selective, risk-based approach is consistent with existing clinical guidelines but cannot be directly established from the present meta-analysis. Current guidelines suggest that fascial closure should be considered particularly in patients and procedures associated with a higher baseline risk of port-site hernia, with three clinical scenarios appearing particularly relevant. The first is the umbilicus and midline, where the linea alba is weak and most hernias appear [5,7]. The second is specimen-extraction and large working-port sites, where the defect is deliberately stretched and which produced the most hernias in the bariatric cohorts here [8,9]. The third is obese and bariatric patients, whose raised intra-abdominal pressure and slower wound healing compound the danger [3,29]. These factors should be regarded as guideline-based clinical considerations rather than quantitative findings of the present meta-analysis.

Whether trocar design influences the benefit of fascial closure remains unclear. Although non-bladed and radially expanding trocars may reduce fascial trauma [13,14,15], the available evidence is limited by differences in hernia detection and small event numbers [5,9,21]. Furthermore, variability in trocar diameter, design, anatomical location, and surgical procedures represents an important source of clinical heterogeneity, limiting the comparability of the included studies and the interpretation of the pooled results. As the available data did not allow reliable subgroup analyses according to these trocar characteristics, no firm conclusions can be drawn regarding their influence on the effect of fascial closure. Although robotic surgery was included in our eligibility criteria, none of the included studies evaluated robotic procedures. This represents an important evidence gap, particularly given the widespread use of robotic surgery, and highlights the need for comparative studies assessing fascial closure in this setting.

Port-site hernia is a time-dependent outcome, and the duration of surveillance in a study determines its measured incidence as surely as the diagnostic modality does. Differences in follow-up duration have two important implications for interpreting our findings. The first concerns statistical power. Fascial defects continue to fail over time. Fink et al. reported an increase in incisional hernia incidence after midline laparotomy from 12.6% at 1 year to 22.4% at 3 years [30]. This time-dependent pattern is further supported by the long-term follow-up of the STITCH randomized trial, in which the cumulative incidence of incisional hernia reached 34% with small-bites and 49% with large-bites fascial closure at 13 years, with a median follow-up of 8 years [31]. Although these findings derive from midline laparotomy rather than trocar sites, they reinforce the principle that short follow-up may substantially underestimate the eventual incidence of fascial failure. Trocar sites behave similarly. A prospective cohort of patients undergoing laparoscopic cholecystectomy, followed clinically and sonographically for three years, identified umbilical trocar-site hernias in approximately one-quarter of patients [32]. Similarly, López-Negrete Cueto et al. identified three additional trocar-site hernias during long-term ultrasonographic follow-up after bariatric surgery, suggesting that longer surveillance may detect hernias not identified at earlier assessments [33]. A study that closes surveillance at six or twelve months therefore does not measure a lower hernia rate; it measures an earlier point on the same accrual curve. Because the events it fails to capture are distributed across both arms, the principal effect is a loss of events and hence of power, which widens confidence intervals and draws borderline effects toward the null. This is readily apparent within the present dataset: the two studies with the shortest surveillance (Shalhav, mean 4.8 months; Singal, 3–6 months) recorded no events at all and were non-estimable, while the shortest randomized trial (Yacoub, 6 months) contributed only five events in total. Consequently, differences in follow-up duration may have influenced the number of detected events and contributed to the uncertainty of the pooled estimate. The second consequence is more serious, because it introduces bias rather than imprecision. Where follow-up duration differs between the closure and non-closure arms of the same study, differential ascertainment arises and the resulting odds ratio no longer estimates the effect of closure alone. This is precisely the mechanism invoked above to explain the null result of Karampinis and colleagues, whose closure arm was followed substantially longer than the comparator and would therefore be expected to accumulate more detected hernias irrespective of any true treatment effect [3]. An analogous concern applies to before/after designs such as that of Rebibo and colleagues, in which the two arms are separated in calendar time and may differ in the duration and completeness of imaging follow-up as well as in the intervention itself [9]. Imbalance of this kind is not captured in any obvious way by the ROBINS-I confounding domain, yet it is capable of reversing the direction of an estimate; we suggest that it be appraised explicitly, alongside detection modality, in future syntheses of this outcome and that it be considered as a candidate domain in any adaptation of ROBINS-I to time-dependent surgical outcomes. Practically, trials of trocar-site closure should report time-to-event data rather than crude proportions, should apply an identical imaging protocol at identical intervals to both arms, and should extend surveillance to at least three years. Differences in follow-up duration and outcome ascertainment may therefore have contributed to the substantial heterogeneity observed in the primary analysis (I^2^ = 72%), although their relative contribution cannot be determined from the available data.

Several limitations should be acknowledged. First, seven of the ten included studies were observational, and four were judged to have a serious risk of bias, primarily because of confounding by indication. In routine practice, fascial closure is often preferentially performed at trocar sites considered to be at higher risk of hernia, such as larger or extraction-site defects, which may underestimate the true benefit of closure. Baseline age and BMI were summarized descriptively using aggregate study-level data. For studies reporting group-specific means, standard deviations, and sample sizes, aggregate means were calculated using sample-size weighting. Aggregate standard deviations were reconstructed by combining within-study variance and between-study differences in group means. These estimates were used exclusively for descriptive purposes, without inferential comparisons, because individual participant data were unavailable and observations were clustered within studies. Second, considerable clinical heterogeneity existed across the included studies, including differences in surgical populations, trocar size and type, anatomical port location, and closure techniques, ranging from conventional fascial suturing to dedicated closure devices. Although a random-effects model was used to account for between-study variability, the overall pooled estimate should be interpreted as a broad summary of heterogeneous evidence rather than as a treatment effect directly applicable to every surgical setting. Third, methods for detecting port-site hernias varied substantially across studies, from clinical examination alone to ultrasonography or computed tomography. Imaging may identify asymptomatic hernias that would otherwise remain undetected, potentially contributing to differences in reported incidence. Although a subgroup analysis according to detection method was considered, the small number of studies, overlapping diagnostic approaches, and differences in follow-up duration precluded a reliable comparison. Fourth, the evidence from randomized controlled trials was limited to three relatively small studies. In accordance with Cochrane recommendations, publication bias was not formally assessed because fewer than ten studies were available. Fifth, surveillance duration and detection modality varied widely across the included studies and were imbalanced between arms in at least one of them, so the pooled estimate reflects differences in ascertainment as well as in treatment. Additionally, the exploratory subgroup analysis by surgical setting was conducted post hoc and combined randomized and observational studies, with only four estimable studies per subgroup. Its limited statistical power, together with incomplete reporting of trocar characteristics and anatomical port location, precluded reliable further subgroup analyses or meta-regression; consequently, the absence of a significant subgroup difference should not be interpreted as evidence of equivalent treatment effects across surgical settings. Finally, screening was restricted to English-language publications, and unpublished studies were not sought, potentially introducing language and publication bias. Corresponding authors were not contacted to obtain missing or clarify ambiguous data, which may have further limited the completeness of the available evidence.

## 5. Conclusions

The primary analysis was inconclusive. Although the point estimate favoured fascial closure, the confidence interval included no effect and between-study heterogeneity was substantial. Evidence from randomized trials remained sparse and imprecise. Although robotic procedures were eligible, none of the included studies evaluated robotic surgery, limiting the applicability of these findings to robotic port sites. A clinically relevant benefit of fascial closure remains possible, but the available evidence is insufficient to establish its magnitude with confidence. Current guidelines support consideration of closure for trocar defects ≥10 mm, particularly at umbilical and specimen-extraction sites; however, these recommendations cannot be directly established from the present meta-analysis. Adequately powered randomized trials with standardized techniques and long-term follow-up are needed to clarify its benefit.

## Figures and Tables

**Figure 1:**
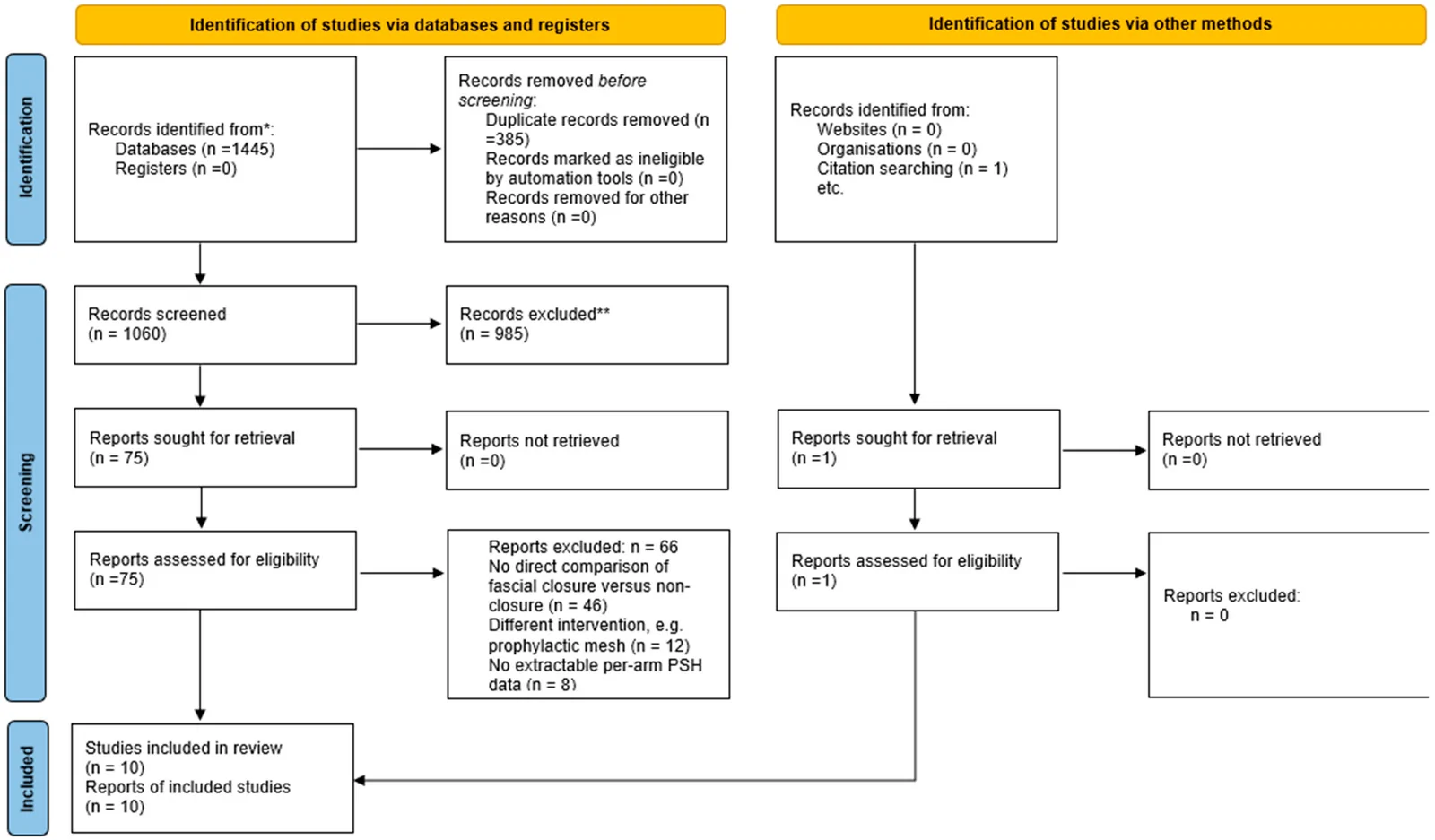
PRISMA 2020 flow diagram.

**Figure 2:**
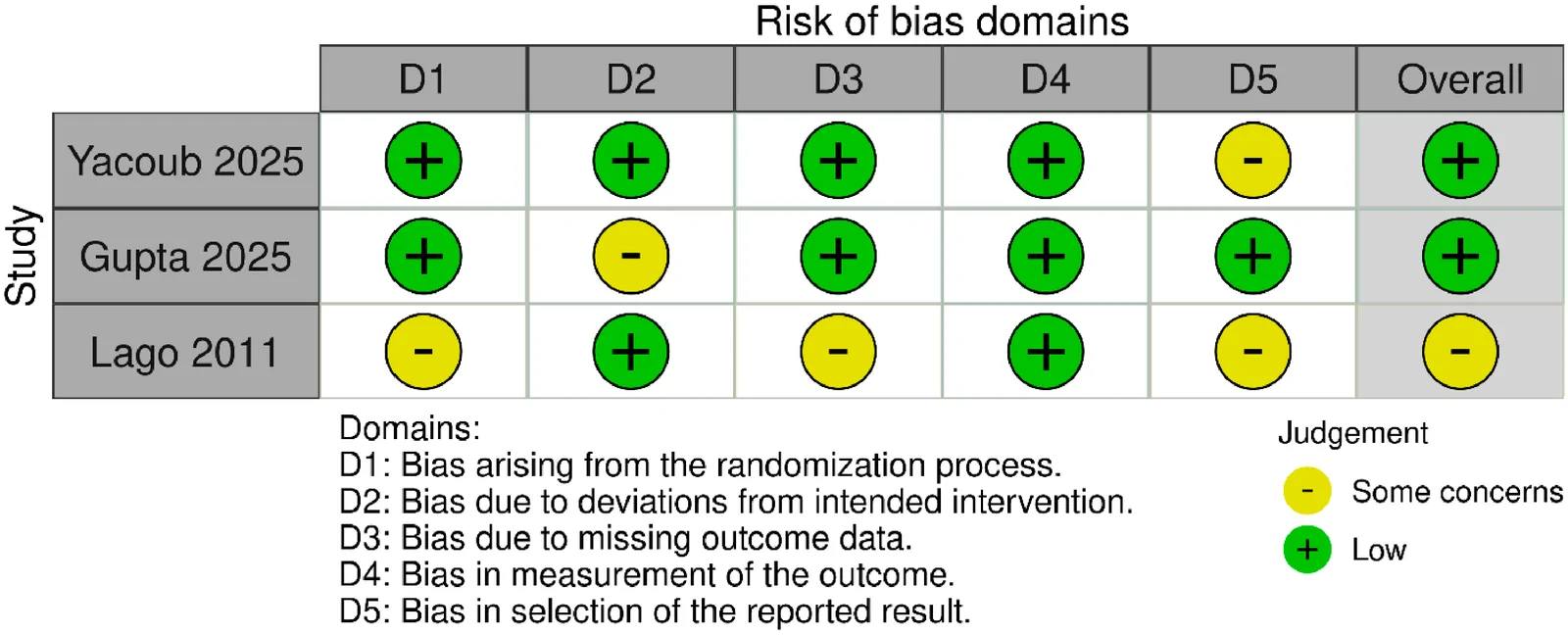
RoB-2 traffic-light plot (RCTs).

**Figure 3:**
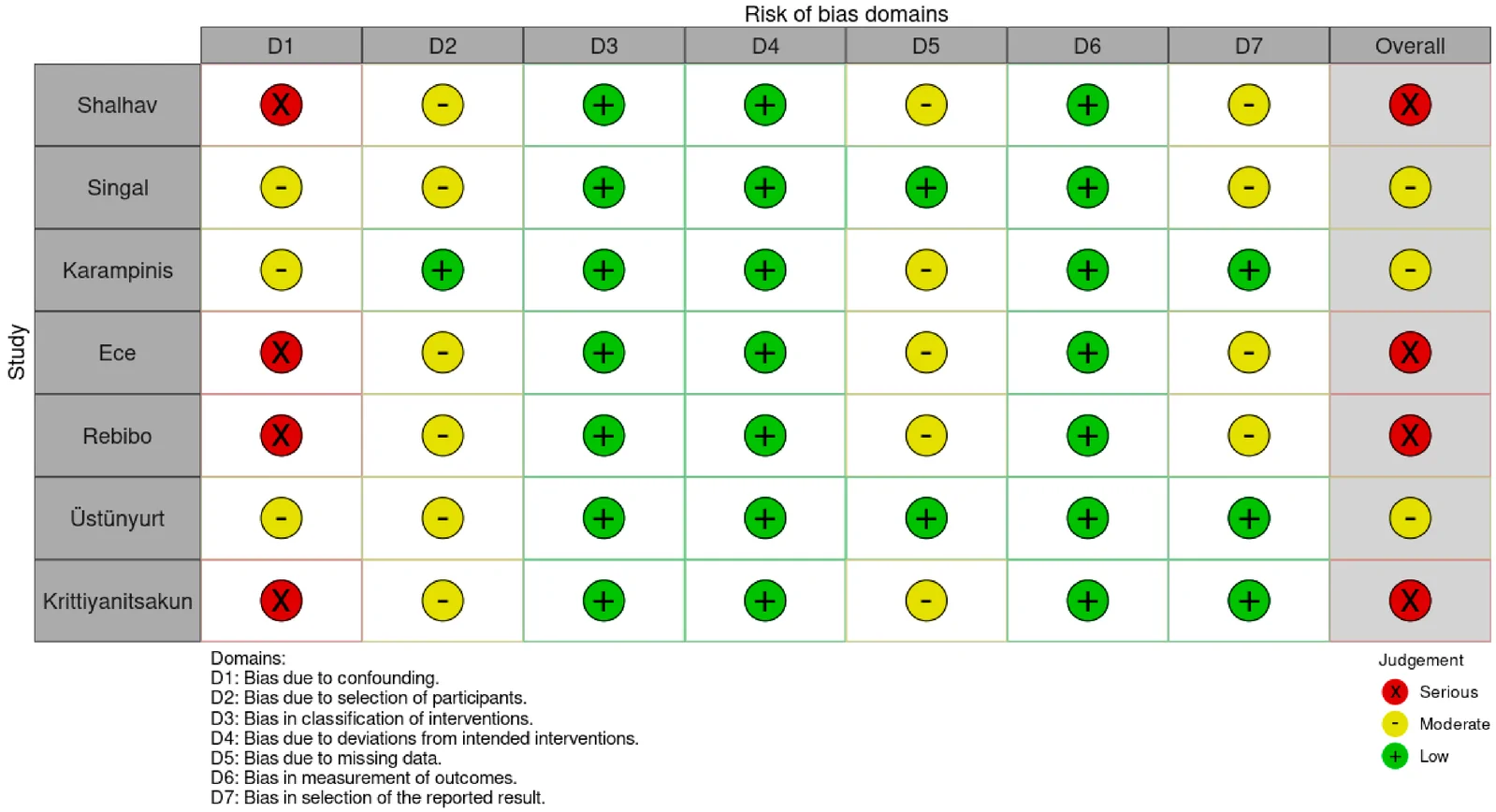
ROBINS-I traffic-light plot (observational studies).

**Figure 4:**
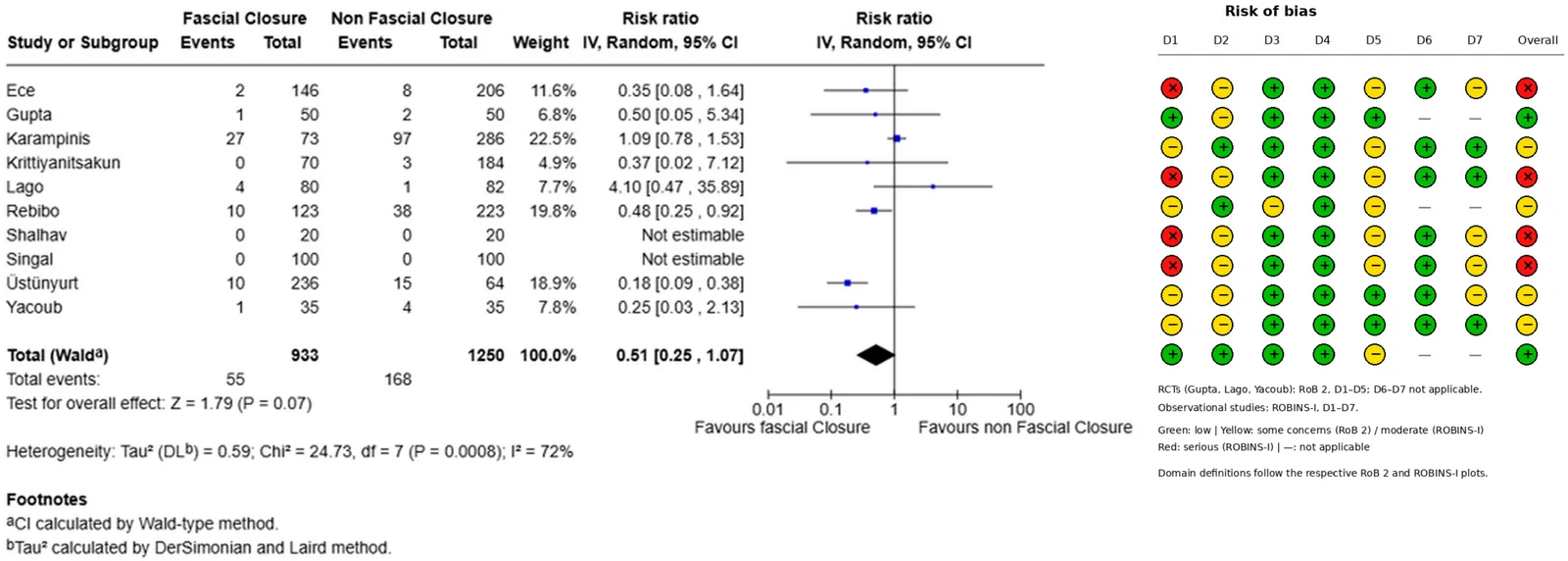
Primary meta-analysis comparing fascial closure versus non-closure for port-site hernia incidence, with study-level risk-of-bias assessments. Risk ratios (RRs) and 95% confidence intervals (CIs) were calculated using a random-effects model. Risk of bias was assessed using RoB 2 for randomized controlled trials and ROBINS-I for observational studies. Green indicates low risk, yellow indicates some concerns (RoB 2) or moderate risk (ROBINS-I), and red indicates serious risk (ROBINS-I). D1–D7 represent the respective risk-of-bias domains; overall judgments are shown in the final column. Domains not applicable to RoB 2 are indicated by dashes.

**Figure 5:**
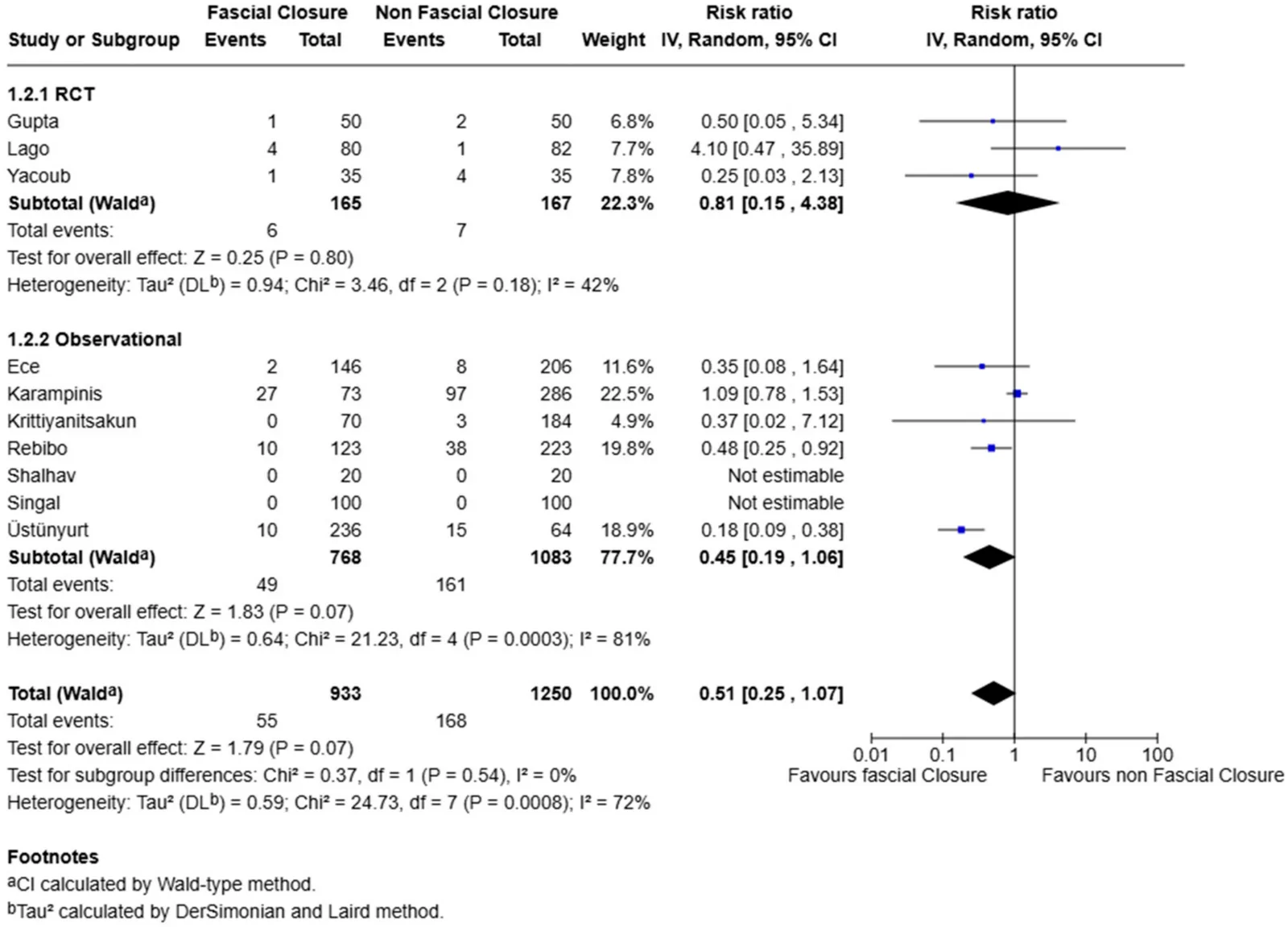
Subgroup analysis comparing fascial closure versus non-closure for port-site hernia incidence, stratified by study design (randomized controlled trials versus observational studies). The test for subgroup differences was not statistically significant (*p* = 0.54).

**Figure 6:**
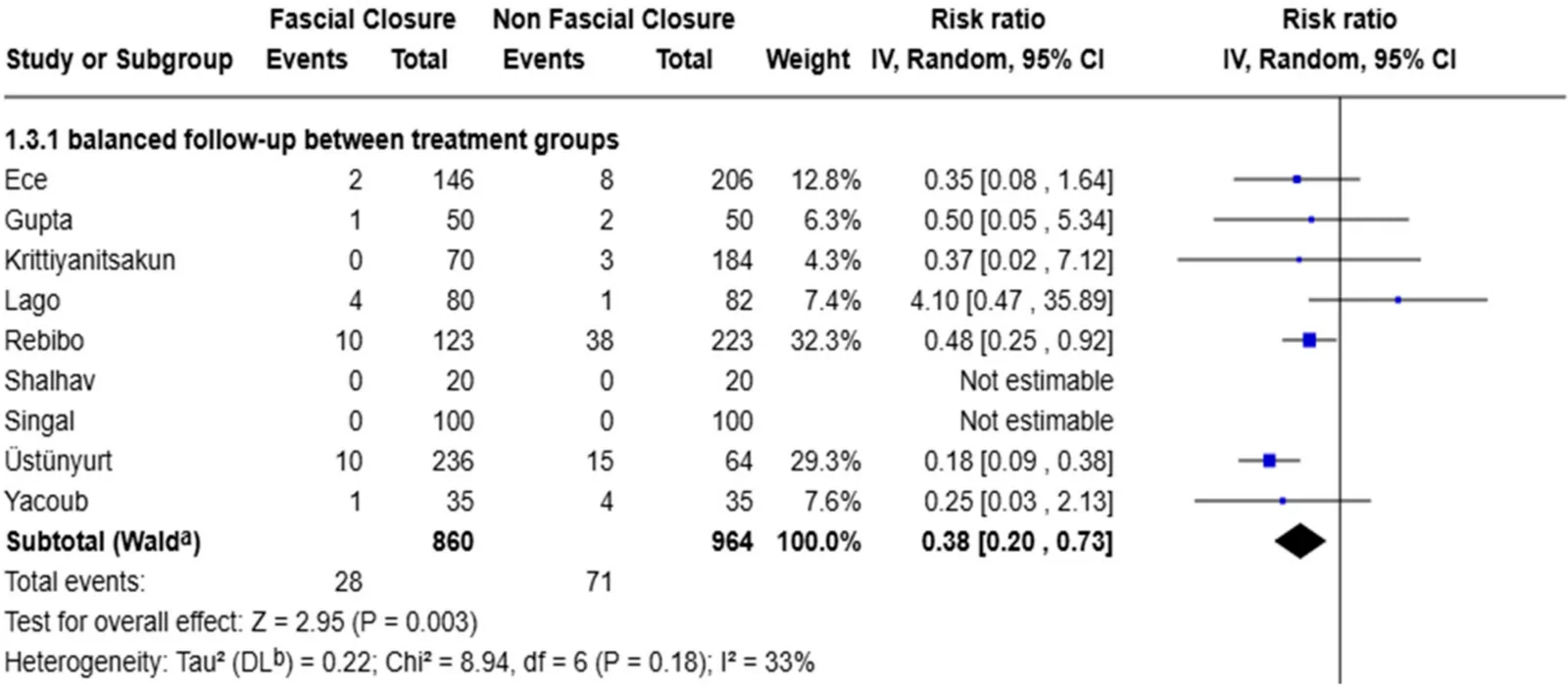
Sensitivity analysis excluding the study with marked between-group imbalance in follow-up duration (Karampinis et al.) [3]. Shalhav et al. [15] and Singal et al. [22] were retained in the analysis but did not contribute to the pooled risk-ratio estimate because both treatment arms reported zero events.

**Table 1:** (**A**) Study design and baseline patient characteristics. (**B**) Trocar characteristics and port-site hernia outcomes.

(**A**)						
**Study (Year)**	**Country**	**Design**	**Surgical Setting**	**C/NC (n)**	**Age (C/NC), Years**	**BMI (C/NC), kg/m^2^**
**Ece (2018)** [8]	Turkey	Retrospective cohort	Sleeve gastrectomy (bariatric)	146/206	35.8 ± 14.6/37.2 ± 15.2	44.9 ± 5.1/45.1 ± 4.9
**Gupta (2025)** [1]	India	RCT	Mixed (cholecystectomy, appendectomy, hernia)	50/50	40.4 ± 10.75/38.58 ± 10.70	NR
**Karampinis (2020)** [3]	Germany	Prospective cohort	Bariatric	73/286	44 ± 10/43 ± 12	51 ± 9/49 ± 7
**Krittiyanitsakun (2023)** [21]	Thailand	Retrospective cohort	Colorectal	70/184	63 ± 12/63 ± 11	23 ± 3/24 ± 4
**Lago (2011)** [2]	Spain	RCT	Mixed (appendectomy, cholecystectomy)	80/82	49/46	NR
**Rebibo (2019)** [9]	France	Prospective before/after	Sleeve gastrectomy (bariatric)	123/223	40.6 ± 11.9/45 (18–68)	45.4 ± 5.8/47.6 (35–75.4)
**Shalhav (2002)** [15]	USA	Retrospective (matched)	Renal surgery	20/20	66 (40–90)/59 (40–86)	26.3 (20.2–33.9)/27.8 (19.3–44.4)
**Singal (2016)** [22]	India	Prospective	Mixed, non-obese	100/100	NR	NR
**Üstünyurt (2020)** [5]	Turkey	Retrospective cohort	Benign gynecologic	236/64	NR	NR
**Yacoub (2025)** [4]	Egypt	RCT	Bariatric	35/35	32.3 ± 9.7/33.3 ± 10.0	48.2 ± 5.5/47.4 ± 5.4
(**B**)						
**Study (Year)**	**Trocar Size and Type**	**Port Location**	**PSH Detection**	**Follow-Up**	**PSH Events (C/NC)**	
**Ece (2018)** [8]	15-mm bladed (10-mm camera; 5-mm)	Upper abdomen	Ultrasound	≥24 mo	2/8	
**Gupta (2025)** [1]	10-mm umbilical	Umbilical 10 mm	Clinical + ultrasound	Mean 14 mo	1/2	
**Karampinis (2020)** [3]	12-mm (5-mm never closed)	Multiple 12-mm ports	Ultrasound	Mean ~36 mo (81 ± 20 vs. 27 ± 18 months)	27/97	
**Krittiyanitsakun (2023)** [21]	12-mm non-bladed	Non-umbilical (12 mm)	CT/cross-sectional	Median ~41 mo	0/3	
**Lago (2011)** [2]	11–12-mm blunt-tip	Midline and lateral (11–12 mm)	Clinical + ultrasound after telephone follow-up	24 mo	4/1	
**Rebibo (2019)** [9]	12-mm bladed (epigastric); 15-mm	Epigastric (12 mm); other sites NR	CT	12 mo	10/38	
**Shalhav (2002)** [15]	12-mm blunt (Optiview)	Lateral rectus/anterior axillary (12 mm)	Clinical	Mean 4.8 mo	0/0	
**Singal (2016)** [22]	10-mm blunt	Umbilical camera port (10 mm)	Clinical	3–6 mo	0/0	
**Üstünyurt (2020)** [5]	10/12-mm cutting	Umbilical (10–12 mm)	Ultrasound	Mean 16 mo	10/15	
**Yacoub (2025)** [4]	12–15-mm working port	Right working port (12–15 mm)	Clinical + ultrasound	6 mo	1/4	

**Table 2:** GRADE (Grading of Recommendations Assessment, Development and Evaluation). Upper panel: observational studies; lower panel: randomized controlled trials (RCTs).

Certainty Assessment							№ of Patients		Effect		
№ of Studies	Study Design	Risk of Bias	Inconsistency	Indirectness	Imprecision	Other Considerations	Fascial Closure	Non-Fascial Closure	Relative Risk (95% CI)	Absolute Effect 95% CI)	Certainty
7	non-randomized studies	serious	serious	not serious	not serious	none	49/768 (6.4%)	161/1083 (14.9%)	RR 0.45 (0.19 to 1.06)	82 fewer per 1000 (from 120 fewer to 9 more)	⊕◯◯◯ Very Low
3	randomized trials	not serious	not serious	not serious	serious	none	6/165 (3.6%)	7/167 (4.2%)	RR 0.81 (0.15 to 4.38)	8 fewer per 1000 (from 36 fewer to 142 more)	⊕⊕◯◯ Low

**Table 3:** Narrative summary of secondary outcomes reported by the included studies.

Study	Outcome	Fascial Closure	Non-Closure	*p*-Value
Gupta et al., 2025 [1]	Surgical site infection	6/50	4/50	0.688
	Hematoma	0	1	NS
Lago et al., 2011 [2]	Surgical site infection	7/80	3/82	0.154
Yacoub et al., 2025 [4]	Surgical site infection	5/35	4/35	0.721
	Bleeding	1/35	3/35	0.303
	Operative time (min)	121	115	0.069
	Postoperative pain	Higher	Lower	<0.001
Karampinis et al., 2020 [3]	Operative time (min)	166 ± 64	110 ± 55	<0.001
Ece et al., 2018 [8]	Surgical site infection	8/146	11/206	NR
	Bleeding	2/146	3/206	NR
	Operative time (min)	54.2 ± 12.3	45.8 ± 11.4	0.124
	Bowel incarceration	One case reported; group unclear	NR	NR
Rebibo et al., 2019 [9]	Wound infection	1 case	NR	NR
	Operative time (min)	58.0	90.2	<0.001
Krittiyanitsakun et al., 2023 [21]	Surgical site infection	Not separately reported	Not separately reported	NS
	Operative time (min)	170	123	0.004
	Postoperative pain	Higher	Lower	0.018
Singal et al., 2016 [22]	Surgical site infection	2	1	NS
	Bleeding	0	0	NR
Shalhav et al., 2002 [15]	Operative time (min)	196	175	0.39
	Analgesic requirement	No significant difference reported between groups	—	0.52
Üstünyurt et al., 2020 [5]	Secondary outcomes	Not reported by closure group	Not reported by closure group	NR

Abbreviations: NR, not reported; NS, not statistically significant. Operative times are in minutes; ± denotes standard deviation where reported. Qualitative findings are retained when numerical group-specific results were unavailable.

**Figure A1:**
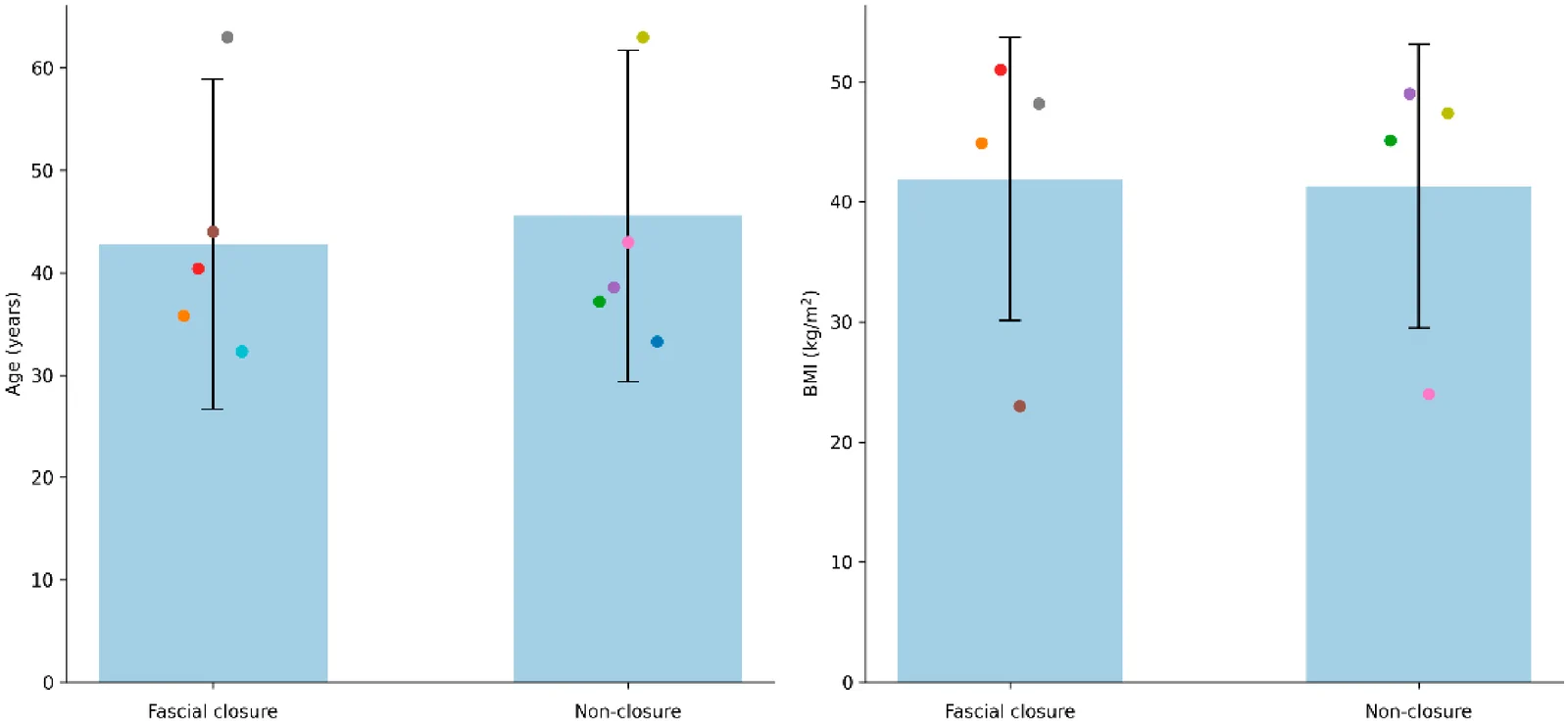
Descriptive comparison of baseline age and body mass index according to fascial closure status. Bars represent sample-size-weighted aggregate means, and error bars represent aggregate standard deviations reconstructed from available study-level summary data. Individual points represent study-specific group means. No inferential statistical comparisons were performed. These aggregate estimates should be interpreted cautiously because of between-study heterogeneity and the absence of individual participant data.

**Figure A2:**
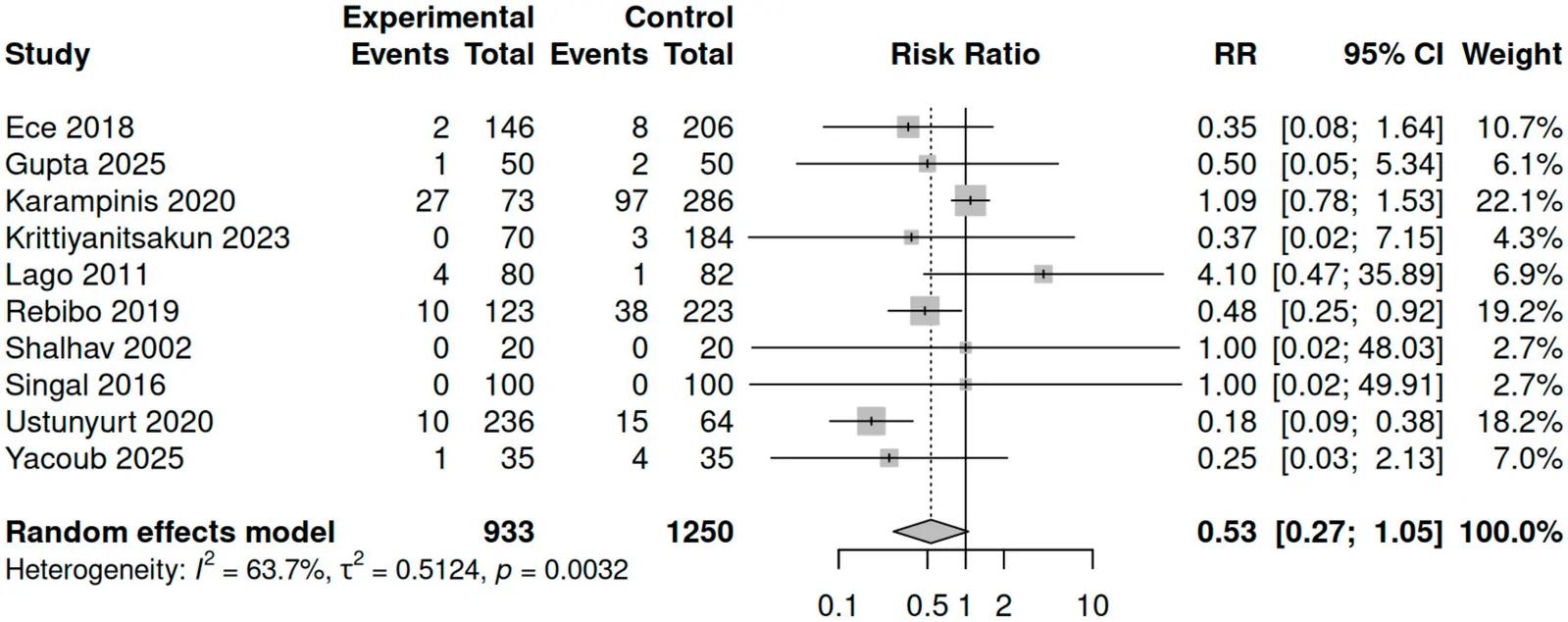
Sensitivity analysis of fascial closure versus non-closure for port-site hernia incidence, including all 10 eligible studies [1,2,3,4,5,8,9,15,21,22]. A continuity correction of 0.5 was applied to zero-event studies. Risk ratios (RRs) were pooled using an inverse-variance random-effects model with DerSimonian–Laird estimation. The forest plot was generated using the meta package in R statistical software through the Posit Cloud (RStudio) platform.

**Figure A3:**
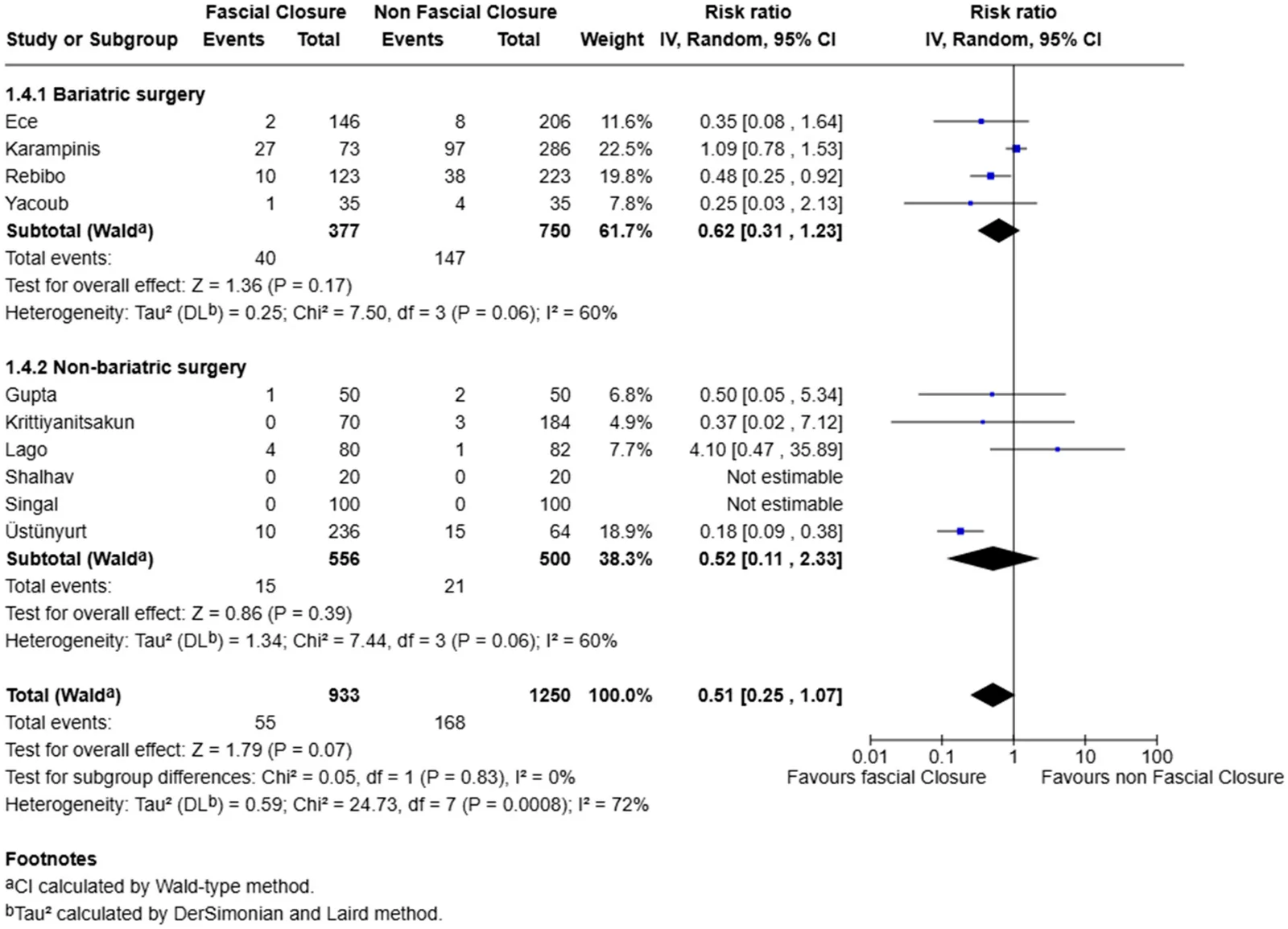
Exploratory post hoc subgroup analysis of fascial closure versus non-closure for the incidence of port-site hernia, stratified by surgical setting (bariatric versus non-bariatric surgery). The forest plot presents study-specific and pooled risk ratios (RRs) with 95% confidence intervals (CIs), calculated using a random-effects inverse-variance model with DerSimonian–Laird estimation of between-study variance. The test for subgroup differences showed no statistically significant interaction (*p* = 0.83). Studies with zero events in both groups were retained in the analysis but did not contribute to the pooled RR estimates.

**Figure A4:**
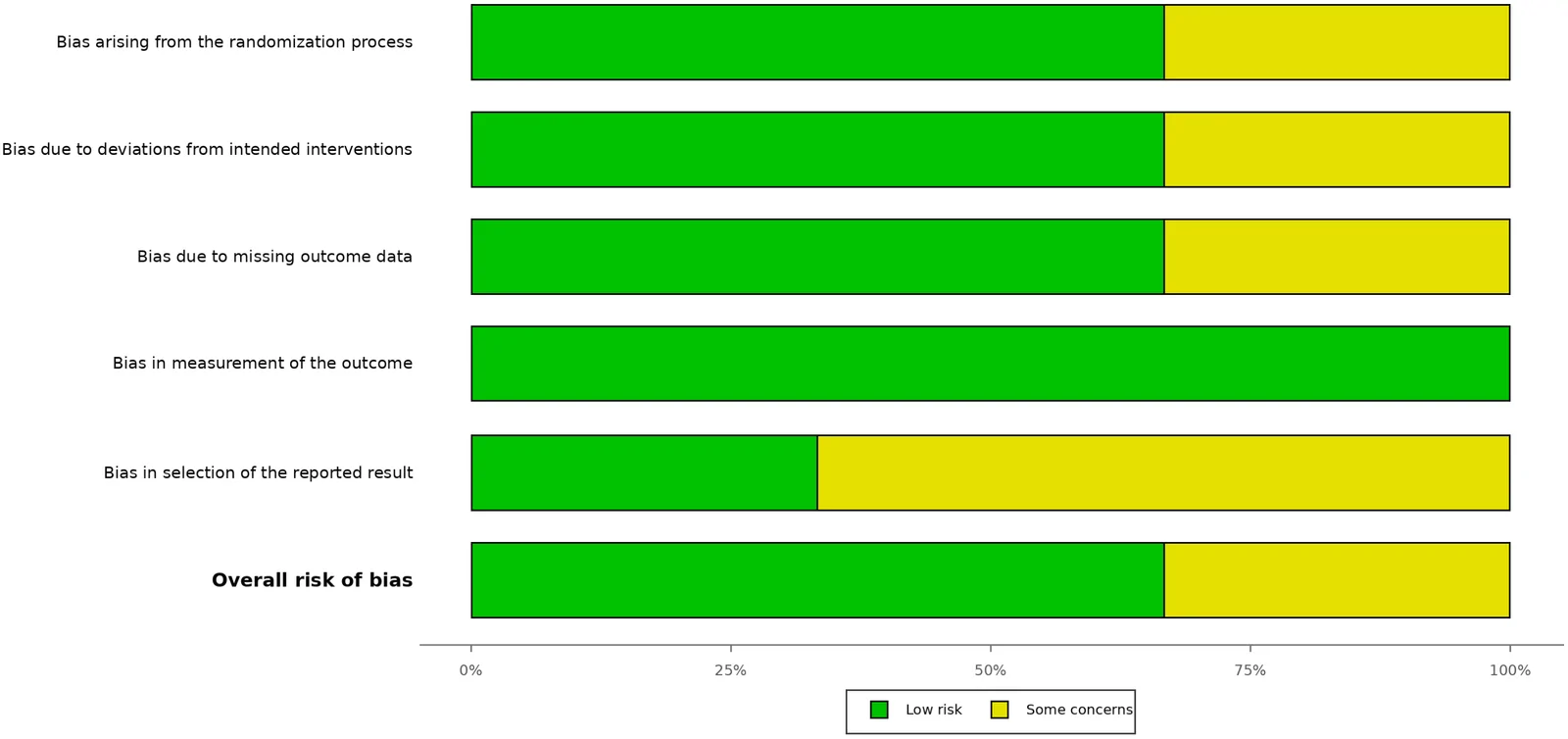
RoB-2 summary plot (RCTs).

**Figure A5:**
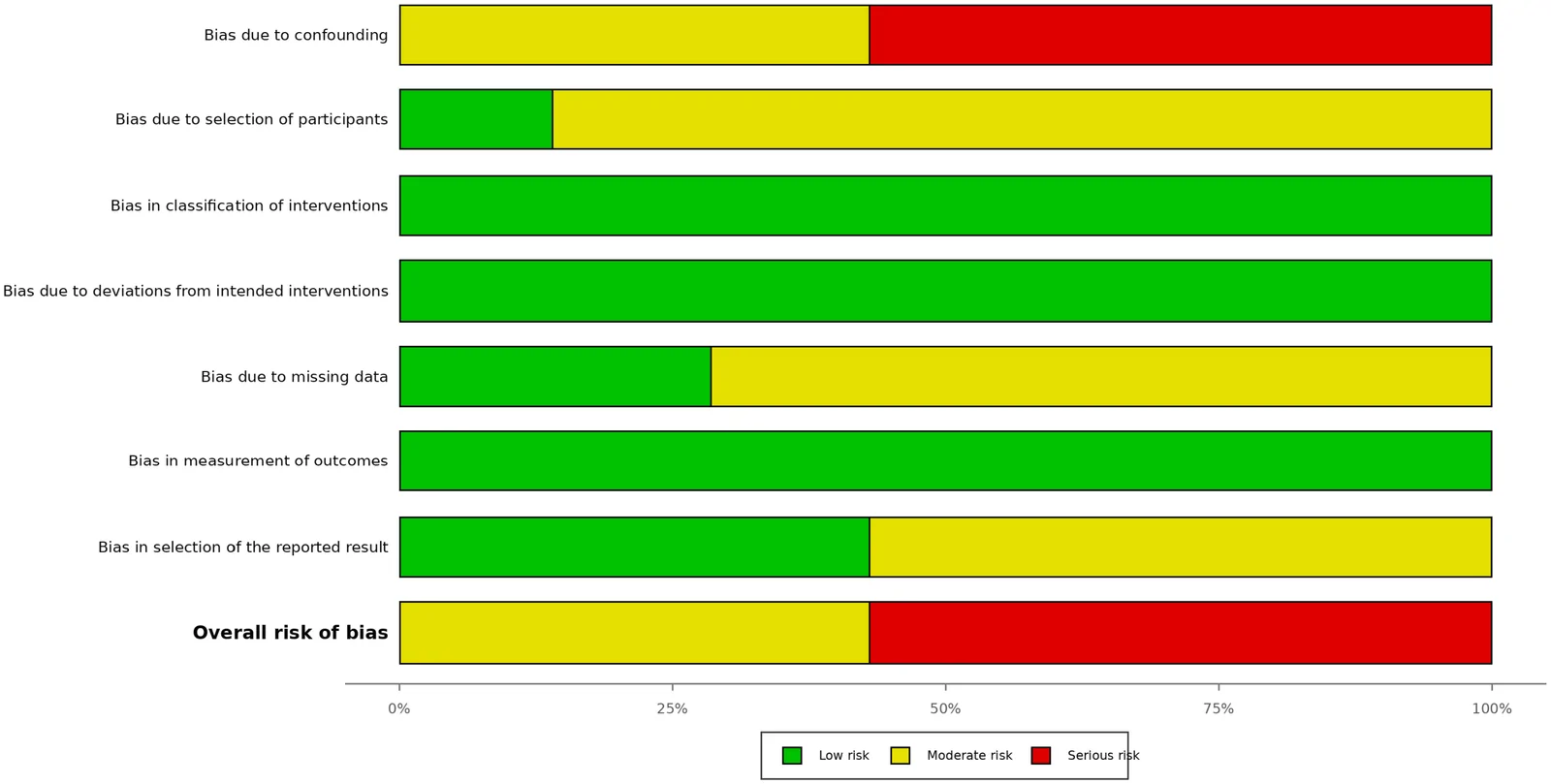
ROBINS-I summary plot (observational studies).

**Figure A6:**
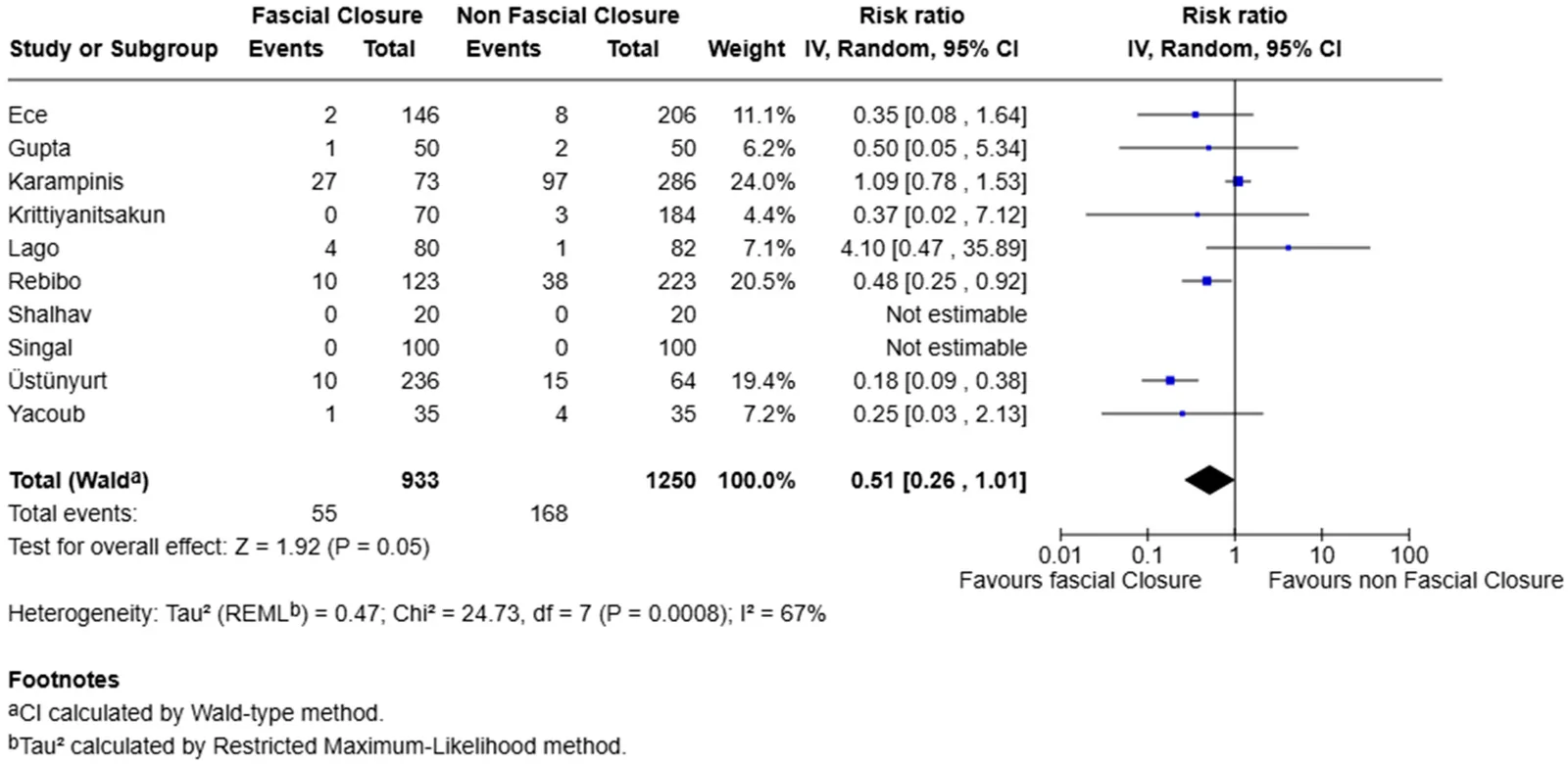
Sensitivity analysis of fascial closure versus non-closure for port-site hernia using a random-effects model with restricted maximum likelihood (REML) estimation of between-study variance (τ^2^). Squares represent study-specific risk ratios (RRs), horizontal lines indicate 95% confidence intervals (CIs), and the diamond represents the pooled effect (RR 0.51, 95% CI 0.26–1.01; I^2^ = 67%). Studies with zero events in both groups were non-estimable.

**Figure A7:**
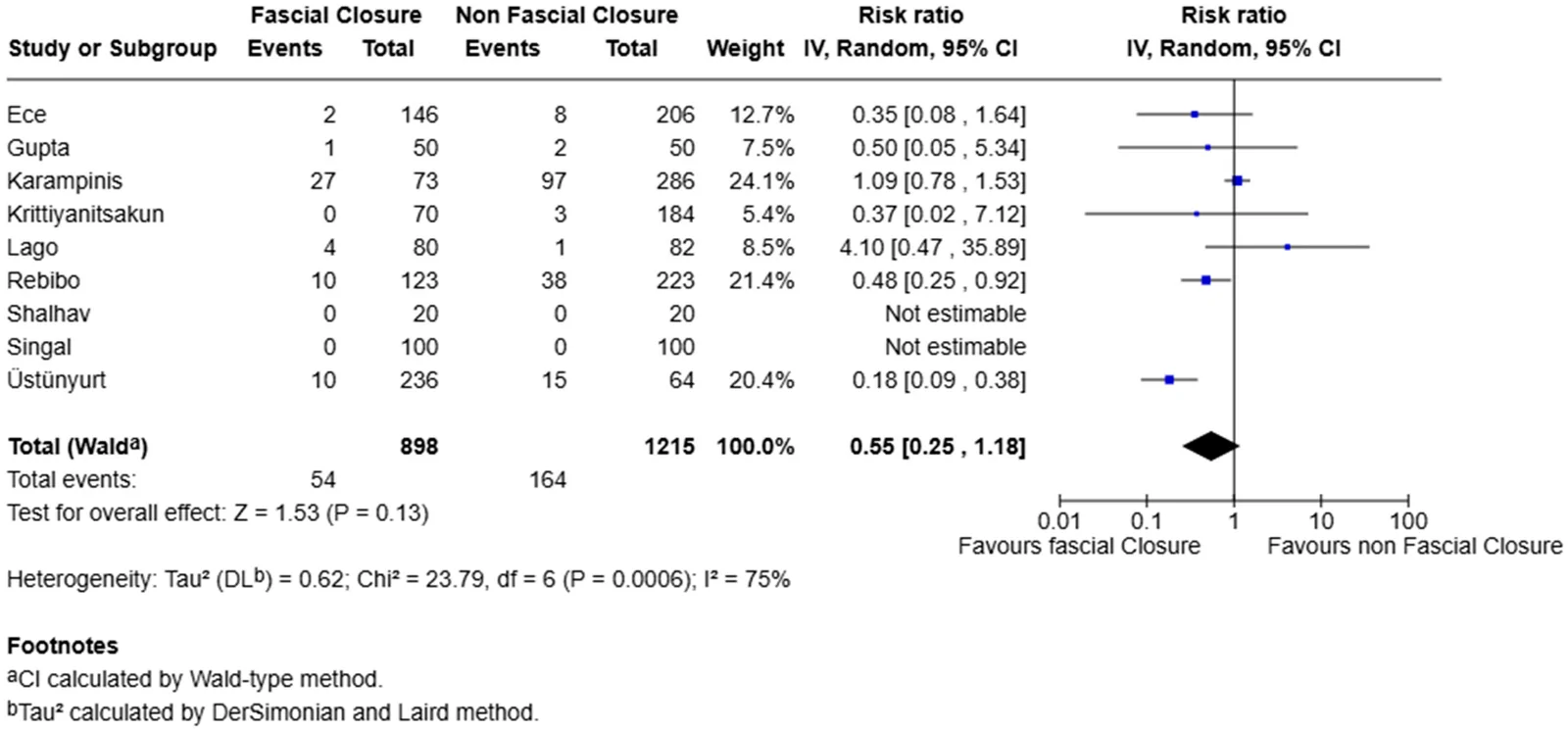
Sensitivity analysis excluding Yacoub et al. [4] to assess the influence of the post-registration amendment to the age eligibility criterion. Risk ratios (RRs) and 95% confidence intervals (CIs) were pooled using an inverse-variance random-effects model with DerSimonian–Laird estimation of between-study variance. The pooled estimate was RR 0.55 (95% CI 0.25–1.18; I^2^ = 75%). The two studies reporting zero events in both groups (Shalhav et al. [15] and Singal et al. [22]) were retained but did not contribute to the pooled RR estimate.

## Data Availability

All data analyzed in this systematic review and meta-analysis were extracted from previously published studies cited in the manuscript. The extracted dataset and data used for the meta-analysis are available from the corresponding author upon reasonable request.

## Supplementary Materials

The following supporting information can be downloaded at: https://www.mdpi.com/article/10.3390/jcm15218280/s1, Table S1: PRISMA 2020 checklist.

## Abbreviations

The following abbreviations are used in this manuscript:

CI	Confidence interval
GRADE	Grading of Recommendations Assessment, Development and Evaluation
PRISMA	Preferred Reporting Items for Systematic Reviews and Meta-Analyses
PSH	Port-site hernia

## Appendix A

**Table A1:** Search Strategy.

#	Query	Results from 7 March 2026
1	(port site hernia or trocar site hernia or incision site hernia or fascial defect or Richter hernia or fascial closure).mp. [mp=ti, ab, hw, tn, ot, dm, mf, dv, kf, fx, dq, cw, bt, nm, ox, px, rx, an, ui, sy, ux, mx]	5286
2	(Laparoscopy or Robotic or da Vinci or Surgical Instruments or Bladeless or 8 mm or radially expanding).mp. [mp=ti, ab, hw, tn, ot, dm, mf, dv, kf, fx, dq, cw, bt, nm, ox, px, rx, an, ui, sy, ux, mx]	515,792
3	1 and 2	1445
4	remove duplicates from 3	1060

**Table A2:** Domain-level risk-of-bias assessment of the seven included observational studies using the Risk of Bias in Non-randomized Studies of Interventions (ROBINS-I) tool. Judgments are presented for each of the seven ROBINS-I domains, together with concise study-specific justifications and overall risk-of-bias ratings.

Study	ROBINS-I Domain	Judgment	Reason for Judgment
Shalhav [15]	D1—Confounding	Serious	Non-closure restricted by patient/port eligibility; non-random allocation.
	D2—Selection of participants	Moderate	20 matched pairs selected from 90 procedures.
	D3—Classification of interventions	Low	Closure and non-closure explicitly documented.
	D4—Deviations from intended interventions	Low	Both techniques described; no major deviations reported.
	D5—Missing data	Moderate	Mean follow-up only 4.8 months; late hernias may be missed.
	D6—Measurement of outcomes	Low	Postoperative hernia assessment described consistently.
	D7—Selection of reported result	Moderate	No registered analysis plan identified; selective reporting cannot be excluded.
	Overall	Serious	Highest domain-level risk determines overall judgment.
Singal [22]	D1—Confounding	Moderate	Non-randomized comparison; procedural and patient factors may confound.
	D2—Selection of participants	Moderate	Patients assigned to groups without clearly described allocation safeguards.
	D3—Classification of interventions	Low	Closure status and blunt-trocar use described.
	D4—Deviations from intended interventions	Low	Interventions appear to have followed the stated techniques.
	D5—Missing data	Low	Reported follow-up of 6–8 months; no clear differential attrition reported.
	D6—Measurement of outcomes	Low	Both groups assessed for port-site complications.
	D7—Selection of reported result	Moderate	No prespecified protocol or analysis plan identified.
	Overall	Moderate	Highest domain-level risk determines overall judgment.
Karampinis [3]	D1—Confounding	Moderate	Non-randomized closure decisions; measured risk factors explored by regression.
	D2—Selection of participants	Low	Prospective cohort with defined invitation to ultrasound follow-up.
	D3—Classification of interventions	Low	Fascial closure technique and eligible port sites specified.
	D4—Deviations from intended interventions	Low	Standardized perioperative care reported.
	D5—Missing data	Moderate	Follow-up duration differed markedly between groups.
	D6—Measurement of outcomes	Low	Follow-up ultrasonography performed by designated examiners.
	D7—Selection of reported result	Low	Defined hernia outcome and reported regression analyses.
	Overall	Moderate	Highest domain-level risk determines overall judgment.
Ece [8]	D1—Confounding	Serious	Closure introduced in a later chronological cohort; temporal confounding possible.
	D2—Selection of participants	Moderate	Retrospective cohort; 34/386 patients not in final follow-up sample.
	D3—Classification of interventions	Low	Consecutive closure and non-closure periods clearly described.
	D4—Deviations from intended interventions	Low	Operative approach and closure technique described.
	D5—Missing data	Moderate	Follow-up exclusions and different observation durations.
	D6—Measurement of outcomes	Low	Scheduled clinical follow-up for port-site hernia.
	D7—Selection of reported result	Moderate	No prespecified analysis plan identified.
	Overall	Serious	Highest domain-level risk determines overall judgment.
Rebibo [9]	D1—Confounding	Serious	Historical versus prospective comparison risks secular confounding.
	D2—Selection of participants	Moderate	Non-concurrent cohorts may differ in recruitment and eligibility.
	D3—Classification of interventions	Low	Closure status defines the compared cohorts.
	D4—Deviations from intended interventions	Low	No major intervention deviations apparent from available study description.
	D5—Missing data	Moderate	Comparability of follow-up completeness requires full-text confirmation.
	D6—Measurement of outcomes	Low	Outcome measurement comparability requires full-text confirmation.
	D7—Selection of reported result	Moderate	Prespecification of outcomes/analysis requires full-text confirmation.
	Overall	Serious	Highest domain-level risk determines overall judgment.
Üstünyurt [5]	D1—Confounding	Moderate	Closure at surgeon discretion; multivariable adjustment reported.
	D2—Selection of participants	Moderate	Retrospective identification with eligibility and recall for assessment.
	D3—Classification of interventions	Low	Umbilical fascial closure status recorded.
	D4—Deviations from intended interventions	Low	No important departures from intended interventions reported.
	D5—Missing data	Low	300 patients underwent follow-up ultrasonography; attrition details should be checked.
	D6—Measurement of outcomes	Low	Ultrasonography used to identify symptomatic and occult hernias.
	D7—Selection of reported result	Low	Risk factors and multivariable analysis reported.
	Overall	Moderate	Highest domain-level risk determines overall judgment.
Krittiyanitsakun [21]	D1—Confounding	Serious	Surgeon-directed closure in retrospective comparison; confounding by indication.
	D2—Selection of participants	Moderate	Single-centre retrospective selection; eligibility may differ by treatment.
	D3 — Classification of interventions	Low	12-mm trocar closure status defined.
	D4—Deviations from intended interventions	Low	No major protocol deviations described.
	D5—Missing data	Moderate	Long-term follow-up available, but differential completeness needs assessment.
	D6—Measurement of outcomes	Low	Cross-sectional imaging used for hernia assessment.
	D7—Selection of reported result	Low	Specified hernia outcome and comparisons reported.
	Overall	Serious	Highest domain-level risk determines overall judgment.
